# The 3D genome and its impacts on human health and disease

**DOI:** 10.1093/lifemedi/lnad012

**Published:** 2023-03-23

**Authors:** Siqi Wang, Zhengyu Luo, Weiguang Liu, Tengfei Hu, Zhongying Zhao, Michael G Rosenfeld, Xiaoyuan Song

**Affiliations:** MOE Key Laboratory of Cellular Dynamics, Hefei National Research Center for Physical Sciences at the Microscale, CAS Key Laboratory of Brain Function and Disease, School of Life Sciences, Division of Life Sciences and Medicine, University of Science and Technology of China, Hefei 230026, China; MOE Key Laboratory of Cellular Dynamics, Hefei National Research Center for Physical Sciences at the Microscale, CAS Key Laboratory of Brain Function and Disease, School of Life Sciences, Division of Life Sciences and Medicine, University of Science and Technology of China, Hefei 230026, China; MOE Key Laboratory of Cellular Dynamics, Hefei National Research Center for Physical Sciences at the Microscale, CAS Key Laboratory of Brain Function and Disease, School of Life Sciences, Division of Life Sciences and Medicine, University of Science and Technology of China, Hefei 230026, China; MOE Key Laboratory of Cellular Dynamics, Hefei National Research Center for Physical Sciences at the Microscale, CAS Key Laboratory of Brain Function and Disease, School of Life Sciences, Division of Life Sciences and Medicine, University of Science and Technology of China, Hefei 230026, China; Department of Biology, Hong Kong Baptist University, Hong Kong 999077, China; Howard Hughes Medical Institute, Department and School of Medicine, University of California, San Diego, La Jolla, CA 92093, USA; MOE Key Laboratory of Cellular Dynamics, Hefei National Research Center for Physical Sciences at the Microscale, CAS Key Laboratory of Brain Function and Disease, School of Life Sciences, Division of Life Sciences and Medicine, University of Science and Technology of China, Hefei 230026, China

**Keywords:** 3D genome, 3C and 3C-based method, 3D genome changes in health and diseases

## Abstract

Eukaryotic genomes are highly compacted in the cell nucleus. Two loci separated by a long linear distance can be brought into proximity in space through DNA-binding proteins and RNAs, which contributes profoundly to the regulation of gene expression. Recent technology advances have enabled the development and application of the chromosome conformation capture (3C) technique and a host of 3C-based methods that enable genome-scale investigations into changes in chromatin high-order structures during diverse physiological processes and diseases. In this review, we introduce 3C-based technologies and discuss how they can be utilized to glean insights into the impacts of three-dimensional (3D) genome organization in normal physiological and disease processes.

## Introduction

In eukaryotes, meter-sized genomic DNA is folded into nanometer-sized nuclei, and chromatin is continuously folded and compacted into complex 3D structures [1]. Distinct chromatin structures are characteristic of different cell states, and the dynamic nature of chromatin structure has been demonstrated during a variety of physiological processes, some of which have been functionally linked to the regulation of gene expression [2–10].

**Figure 1.**
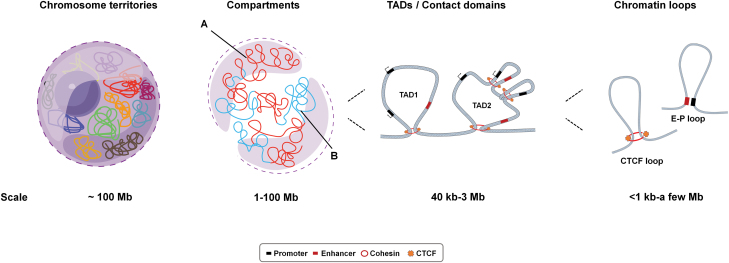
Hierarchical genome organization. Taking an interphase cell as an example and moving from the scale of megabases down to <1 kb, the chromatin in the nucleus is not arbitrarily distributed; rather, each chromosome tends to occupy a characteristic position, which is termed the chromosome territory. For a single chromosome, two so-called “compartments” can be defined by the principal component analysis of the Hi-C data: the A compartments refer to regions of loose chromatin that have higher gene densities and active transcription, and the B compartments refer to compressed chromatin regions with low gene density and inactive transcription. With increased resolution, regions that display extensive self-interactions can be detected on the chromosome, as the chromosome structural units, such as TADs, contact domains, or loop domains, are separated from the adjacent area by boundaries to form an independent regulatory unit. At a finer scale, the contact between regulatory elements (enhancers and promoters) or CTCF loops can be identified.

Studies on 3D genome organization in many species and different cell types clearly show that the chromatin of many eukaryotes is hierarchically organized on different scales in the nucleus (Fig. 1) [11, 12]. Loci on the same chromosome tend to gather together, forming chromosome territories at ~100 Mb [13, 14]. Individual chromosomes can be divided into two different compartments, the A/B compartments, in the 1–100 Mb range [13]. Each chromosome is also composed of multiple topologically associating domains (TADs) in the 40 kb to 3 Mb range. The DNA interaction frequency is much higher inside than outside a TAD. TAD boundaries separate two TADs and hinder communication between different TADs [15, 16]. At 1 kb to a few Mb range are the chromatin loops (including CTCF loops and promoter-enhancer loops) [17, 18]. Mediated by chromatin loops, regulatory elements can interact with genes to form a complex regulatory network [19, 20].

The genome folds through at least two different mechanisms [21]. One is the loop extrusion model [22, 23], where cohesin loads on chromatin and forms a small loop that then extrudes on the chromatin and stops when it encounters two convergently oriented CTCF. The CTCF homodimer can mediate the long-range interaction between different regions of the chromatin to help the formation of loops and TADs, which are likely established via interactions among multiple loops [21, 24]. The enhancer-promoter loop, another type of chromatin loop, is the functional unit of gene regulation [25, 26]. YY1 can also form homodimers to promote the interaction of distal chromatin regions, and YY1 mainly carries out the connection between promoter and enhancer [27]. Other proteins also play roles in genome folding, such as Nipbl (cohesin loading factor) [21], proteins in the mediator complex [28], and recently identified cell lineage-specific master transcription factors that mediate the development of tissue-specific 3D genomes, such as MyoD in muscle development and Pax6 in nervous development [29, 30]. Additionally, RNA also functions in 3D genome organization, exemplified by well-known long noncoding RNAs (lncRNAs) such as *Xist*, *Kcnq1ot1*, and *Firre* [31–33]. More such lncRNAs (*UMLILO*, *ELEANORS*, *HOTAIRM1*, *HOTTIP*, and *CCAT1-5L*) have been identified [34–38], and both the Guttman group and our group have proposed a 3D transcriptional regulation model to further study RNA roles in development and disease [39, 40].

However, the formation of the compartment may depend on phase separation. This is the other mechanism for genome folding, as chromatin with similar properties tends to interact with each other to form separate compartments [21]. The phase-separated compartmental environment is hypothesized to facilitate TAD formation. This compartment-guide hypothesis is supported by the observation of TADs inside compartments and the examination of temporal dynamics in X chromosome reactivation [41]. The role of phase separation in the dynamic changes in higher-order chromatin structures has also been reported recently [42–45].

In this review, we will introduce different methods continuously developed to study the 3D structure of chromatin and present changes in different chromatin structures in normal physiological processes and under diseases, including cancer, congenital diseases, and viral infection.

## Methods for studying the three-dimensional structures of chromatin

Currently, three main approaches are used to investigate the three-dimensional (3D) structures of chromatin: sequencing-enabled, imaging-based, and modeling approaches. In this section, we will overview sequencing-enabled methods and briefly discuss imaging-based methods at both the bulk and single-cell levels. We will not discuss modeling approaches that have been reviewed elsewhere [46, 47].

**Figure 2.**
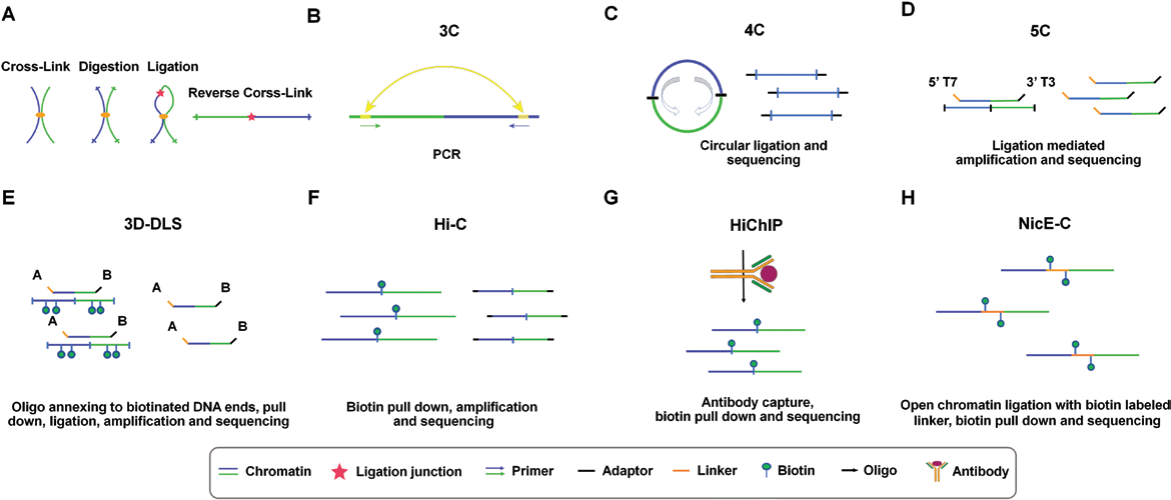
The chromosome conformation capture technique (3C) and 3C-based methods. **(A)** The initial steps—common to all 3C-based methods—include the following: a biological sample (e.g., culture cells or tissue dissected into single-cell suspension, or even homogenized whole organism—for those lower organisms) is crosslinked, followed by digestion with (a) suitable enzyme(s), after which the adjacent DNA ends are connected using a ligase. (**B)** For the simplest 3C, PCR is performed to verify the interaction between two loci of interest based on user-designed primers. (**C)** In 4C, the chromatin containing the targeted DNA sequence is cut by a 2nd restriction enzyme and cyclized, and then the targeted chromatin sites are amplified and sequenced. (**D)** In 5C—building on the basic 3C concept—a universal linker is added to the 3C primer end, and the universal primer is used for PCR amplification, after which samples are subjected to sequencing. (**E)** In 3D-DLS, biotin is added to the 3C library, followed by oligo annealing and ligation, after which the ligation products are captured by streptavidin and finally amplified and sequenced. (**F)** In Hi-C, after restriction enzyme digestion, the ends are supplemented with biotin-labeled deoxynucleotides and ligated together, sonicated to break into smaller DNA fragments, and then subjected to biotin/streptavidin pull down, PCR amplification, DNA size selection and deep sequencing. (**G)** HiChIP. HiChIP performs chromatin crosslinking, digestion, biotin labeling, and proximity ligation following *in situ* Hi-C. After ligation, the nuclei are lysed and disrupted by ultrasound, and then the ChIP experiment is performed with specific antibodies. After obtaining the DNA‒protein complex, DNA elution, and reverse crosslinking are performed, followed by biotin/streptavidin pulldown, library generation, and deep sequencing. (**H)** In NicE-C, nicking enzymes replace the restriction enzymes for chromatin cleavage. The chromatin is repaired at the end, and the dA tail is added. Then, a biotin-labeled bridge linker is added for ligation. Finally, DNA fragments are enriched by biotin and deep sequenced.

Sequencing-enabled methods include ligation-based methods [mainly chromosome conformation capture (3C) and 3C-based technologies] and ligation-free methods. In 3C-based methods, the cells are crosslinked, digested with (a) suitable enzyme(s), and ligated to the adjacent DNA ends (Fig. 2A). Polymerase chain reaction (PCR) is used to detect one-to-one interactions in 3C [48] (Fig. 2B). One-to-all interactions can be detected through circle formation in 4C [49, 50] (Fig. 2C). 5C technology, analogous to 3D-DSL, can detect many-to-many interactions [51, 52] (Fig. 2D and 2E). Hi-C and *in situ* Hi-C are able to capture genome-wide chromatin interactions (all-to-all) [13] (Fig. 2F). Additionally, chromatin-interaction analysis with paired-end tag sequencing (ChIA-PET) combines ChIP-seq and Hi-C to capture the genome-wide interactions mediated by proteins of interest [53]. Similarly, PLAC-Seq and HiChIP can obtain such interactions faster, more sensitively, and cost-effectively [54, 55] (Fig. 2G). Further developed Hi-C methods include increasing resolution to the nucleosome level by using micrococcal nuclease (Micro-C) [56] or focusing on interactions within targeted genome regions. The latter includes Capture Hi-C by designing capture probes specifically targeting certain genome areas [25] or enriching interactions in open chromatin regions (Trac-looping [57], OCEAN-C [58], HiCAR [59], and NicE-C (Fig. 2H) [60]). Additionally, BLHi-C, DLO Hi-C, SAFE Hi-C, and DNase-C are all derivatives of Hi-C [61–64].

For ligation-free approaches, there are split-pool recognition of interactions by tag extension [65], genome architecture mapping [66], and chromatin-interaction analysis via droplet-based and barcode-linked sequencing (ChIA-Drop) [67], which can detect chromatin interactions involving three or more genomic loci that are rarely detected in 3C-based methods.

Beyond sequencing-based approaches, imaging methods combining DNA FISH and microscopy are also used in 3D genome organization studies. Stimulated emission decomposition [68], photoactivated localization microscopy [69], stochastic optical reconstruction microscopy [70], and recent minimal photon fluxes (MINFLUX) [71] appear one after another, breaking through the resolution limitation of conventional light diffraction and even achieving 1–2 nm single molecule positioning accuracy (MINFLUX). OligoFISSEQ [72] and MERFISH [73] solve the problem of a limited number of fluorescent channels. High-resolution and high-throughput imaging methods are also used to detect the interactions of selected genomic regions in individual cells and reveal similar chromatin structures at multiple scales [73–76]. In addition, 3D genome structure and gene transcriptional regulation can be linked in the same single cell by imaging [77]. However, it also has its own limitations, and only a few thousand sites can be detected.

For studying the single-cell 3D genome, several single-cell Hi-C (scHi-C) technologies have also been developed [2, 78–80], with recent high-resolution technologies such as scSPRITE [81] and Dip-C [82]. However, these scHi-Cs have difficulty differentiating different cell subtypes. Several research groups, including ours, are actively developing new methods to achieve single-cell level Hi-C that can distinguish heterogeneous cell subtypes. With these methods, such as single-nucleus methyl-3C sequencing [83], scMethyl-Hi-C [84], and the ones we are developing, it is possible to explore dynamic 3D genome structure changes in different biological processes or disease development at the real single-cell level. Efforts can also be made to combine microimaging approaches, such as MERFISH and MINFLUS [85, 86], and Hi-C-based sequencing approaches to achieve nanoresolution spatial (in tissue) 3D genome detection.

With the development of sequencing-based techniques, bioinformatics tools to analyze sequencing data, including processing of raw data, generation, and normalization of the Hi-C contact map, detection of TADs and chromatin interactions, visualization, and annotation, have been developed accordingly. HiCNorm [87], ICE [88], caICB [89], HiCcompare [90], and Binless [91] are related to Hi-C data standardization. Means for TAD analysis and detection include 3DNetMod [92], HiCDB [93], deDoc [94], DeTOKI [95], Arrowhead [96], TADtree [97], ClusterTAD [98], and SuperTLD [99]. Software for detecting loops includes FitHiC2 [100], HiCCUPS [17], RefHiC [101], LASCA [102], and cLoops [103]. Data visualization browsers also occur, such as WashU Epigenome Browser [104], 3DIV [105], Juicebox [106], and HUGIn [107]. 3Disease Browser is the first disease-centric browser [108], and the recent new web EagleC can more comprehensively identify genome structural variation (SV) [109], while the TADeus2 server can quantify and sort SV pathogenicity [110]. HiCnv and HiCtrans can identify large-scale copy number variation (CNV) and translocations in the genome from Hi-C data [111]. CNVxplorer can display CNVs related to rare diseases and clinical information [112]. Later algorithms with similar functions, such as HiNT, can be recognized at the single-cell level [113]. Related methods have been developed to identify chromatin structures from scHi-C [83, 114]. Different analysis methods have been compared and reviewed nicely elsewhere [115, 116].

## 3D chromatin structural changes in biological processes

The 3D organization of the genome is highly dynamic in normal biological processes. Here, we review the chromatin organization dynamics during mitosis, meiosis, early embryonic development, and cell senescence.

**Figure 3.**
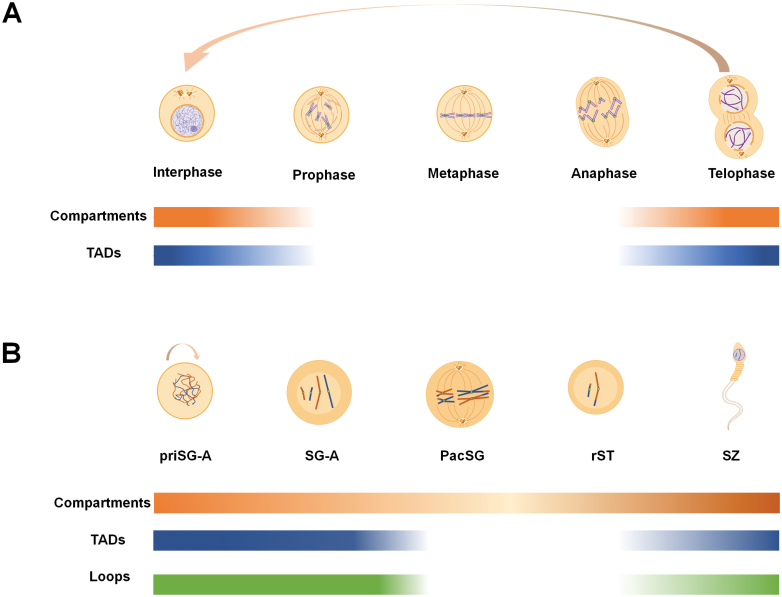
3D structural changes in chromatin in normal development and physiological processes. **(A)** Reprogramming of A/B compartments and TADs during mitosis. At the point when cells enter prophase, very few (if any) large-scale spatial structures (e.g., compartments, TADs) can be detected. In contrast, after cells enters interphase from division, both compartments and TADs reappear. **(B)** Reprogramming of compartments, TADs, and loops during spermatogenesis. Whereas pachytene spermatocyte stage cells have few chromatin structures (decreased compartment strength, loss of TADs, and loops), both compartment strength, TADs, and loops become evident in the sperm at the end of spermatogenesis/gametogenesis [with these structures at levels similar to primitive type A spermatogonia (priSG-A)].

The dynamic 3D genome during mitosis was first described, showing that both chromatin compartments and TADs are disrupted in mitotic prometaphase [117]. CTCF was later found to lose its binding to chromatin during mitotic prometaphase [118], and chromatin structure also changes from G2 phase to metaphase, simulating the process of chromatin aggregation into chromosomes [119]. A more detailed study revealed that when the cell enters interphase again from mitosis, the compartment reappears quickly, which can be detected in anaphase or telophase, and smaller sub-TADs appear earlier and faster than larger topology domains, which appear based on sub-TADs [7, 119] (Fig. 3A).

In mice, TADs are reorganized during mouse spermatogenesis, disappear in meiosis (pachytene stage), and reappear after exiting meiosis (Fig. 3B) [9]. In monkeys, TADs also become very weak at the pachytene stage [120]. A/B compartments are maintained in meiotic prophase [9, 120–122], and meiotic DNA breaks and interhomolog crossovers preferentially form in the gene-dense A compartments [122]. In addition, transcription-related unique compartments are displayed during pachytene, and A/B compartment switching is closely related to meiosis-specific mRNA and piRNA expression [9, 120, 123]. In addition, transcriptionally active and inactive genomic regions form alternating domains consisting of shorter and longer chromatin loops, respectively [124].

Chromatin organization also changes dramatically during embryonic development. In mouse full-grown oocytes, classical TADs are weak, and there are no classical compartments; instead, a special type of polycomb-associated domain appears [125]. On the other hand, mature mouse sperm have classical compartments and TADs, while mature human sperm have no TADs [126]. Upon fertilization, the 3D structure of the parents’ genome is rapidly disassembled, and the zygote chromatin is in a highly loose state. Before early embryo implantation, mature A/B compartments are gradually segregated [2, 6, 127]. TADs are mainly formed at the early embryonic 8-cell stage in humans, yet they are established from the early embryonic 2-cell stage in mice [6, 126, 128].

Cell replicative senescence (RS) is due to the depletion of cell replication capacity [129]. In this process, the 3D structure of chromatin changes accordingly, and genes involved in the cell cycle, which are downregulated, switch from compartment A to B [130]. In addition to RS, there is another senescence, stress-induced premature senescence (SIPS) [131]. SIPS can be divided into two types: oncogene-induced senescence (OIS) and drug-induced cell senescence. In OIS, loss of interaction in heterochromatin, depending on lamin-related proteins, is observed [132]. In addition, senescence-associated heterochromatin domains (SAHDs) can form senescence-associated heterochromatin foci, which bring the active genes located adjacent to SAHDs in the genome in close spatial proximity and promote their expression [133]. A recent study also showed that H3-specific demethylase KDM4-mediated epigenetic modification causes genome-wide 3D spatial changes and chromatin topological remodeling in senescent cells, culminating in persistently high expression of the senescence-associated secretory phenotype during cellular senescence [134]. In drug-induced cell senescence, abnormal gene expression leakage occurs in regions of heterochromatin loss, and facultative heterochromatin tends to switch from the B compartment to the A compartment [135]. Similar chromatin compartment changes are observed in senescent human mesenchymal progenitor cells [136]. Overall, the stability of heterochromatin is crucial for cell senescence, and its loss may lead to premature aging *in vivo* [137, 138].

## 3D genome disorganization in cancer

Cancer is one of the leading causes of death worldwide. 3D genomic alterations may occur at different scales in cancer genomes. In this section, we will discuss the relationship between chromatin structure changes and cancers.

**Figure 4.**
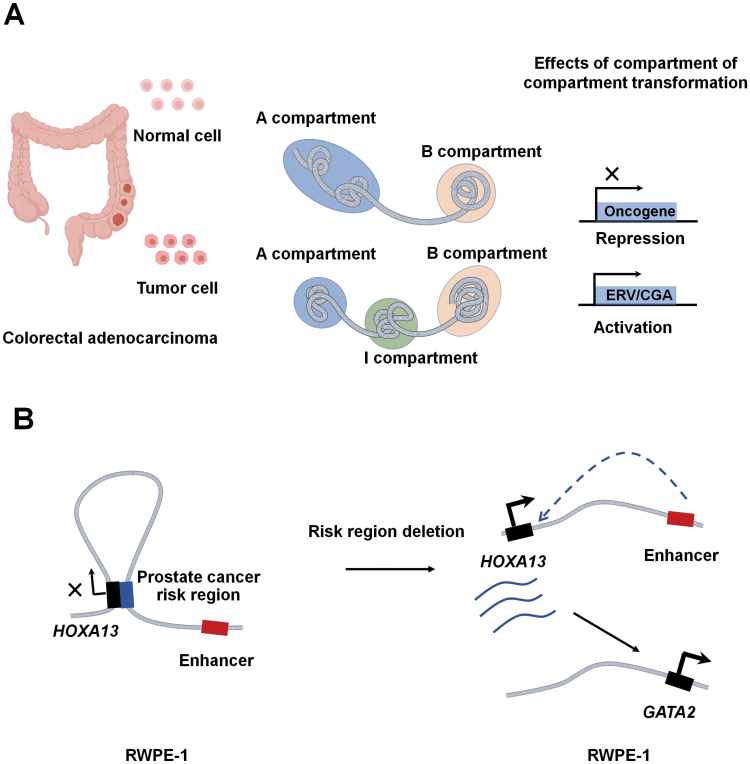
Genome disorganization in cancer. **(A)** Hi-C, RNA-seq and other analyses on colorectal adenocarcinoma and normal tissues show that a part that should exist in compartment A occurs between compartments A and B, which is called compartment I. Subsequent comparison of the expression levels of genes in compartments B and I in tumor and normal cells reveals that a small number of genes are upregulated, including cancer germline antigens (CGAs) and endogenous retroviruses (ERVs), while downregulated genes are enriched in pathways related to Wnt signaling, EMT, invasion, and metastasis functions and are closely related to cancer growth. (**B)** In human prostate epithelium cell (RWPE-1), the *HOXA13* gene is located in a repressive loop. The risk region of the loop anchor is deleted in prostate cancer, and *HOXA13* expression is upregulated, resulting in overexpression of prostate cancer-associated oncogene (*GATA2*).

The recent Pan-Cancer Analysis of Whole Genomes proposes the most comprehensive analysis of cancer genomes to date [139]. On a larger scale, gene-rich mini-chromosomes (chr16 to chr22) have reduced interaction frequencies in MCF-7 breast cancer cells compared to the normal epithelial cell line MCF-10A, which is associated with increased expression of tumorigenesis-related genes [140]. Chromatin state analysis of colorectal adenocarcinoma samples and normal tissue reveals an intermediate region (I compartment) that is structurally distinct from the classical A and B compartments. Extensive changes occur in the spatial division, nuclear localization, and epigenetic status of the B and I compartments. These regional changes are associated with tumor suppressor expression programs associated with reduced cancer risk and improved prognosis [141] (Fig. 4A). In lung adenocarcinoma (LUAD), the main change in the 3D genome structure is the compartment A to B change, and 33.6% of this switch occurs on chromosome 3 harboring the *NCEH1*, *NXPE3*, *MB21D2*, and *DZIP1 L* genes, which may be closely related to the development of LUAD [142]. In prostate cancer, the androgen receptor genes *WNT5A* and *CDK14* and 48 other gene clusters switch from the B to A compartment, indicating that loose chromatin structure promotes the development of pancreatic cancer [143].

On a smaller scale, disruption of the TAD boundary can result in the interaction of otherwise uncontacted enhancers and genes, enabling ectopic activation of genes. This is related to “enhancer adoption” in cancer, the overexpression of oncogenes caused by enhancers and cancer-related genes. For example, disruption of a TAD boundary in T-cell acute lymphoblastic leukemia [144] and gain-of-function *IDH* mutations (thus destroying TADs) in glioma [145] both lead to the activation of proto-oncogenes. In another highly aggressive type of brain tumor (medulloblastoma), prominent oncogenes are overexpressed by enhancer “hijacked” [146]. Chromosome rearrangements can also utilize enhancer hijacking as a driver for *CCNE1* and *IGF2* expression in gastric cancer [147]. Similarly, 3D genome analysis in myeloid leukemia uncovers “silencer hijacking,” where hijacked genes are silenced [148].

In colorectal cancer, tandem duplications containing TAD boundaries are found to lead to *de novo* 3D contact domain formation between the cancer-associated locus (*IGF2*) and super enhancers in the preexisting TADs, resulting in >250-fold overexpression of *IGF2* [149]. Moreover, the spatial location of the genome influences the choice of chromosomal translocation locations [150]. For example, in prostate cancer, androgen signaling induces proximity of the 5ʹ untranslated region of *TMPRSS2* and erythroblast transformation-specific transcription factor genomic loci, leading to subsequent gene fusions [151]. The *MYC*, *BCL*, and *immunoglobulin* loci, which are repeatedly translocated in B-cell lymphomas, are also preferentially located in close spatial proximity to each other [152].

Similar pathological phenomena occur when the enhancer-promoter loop changes. In epithelial carcinoma, the transcription factor KLF5 can activate cancer-related genes through chromatin loops [153]. In the normal human prostate epithelium cell line RWPE-1, the *HOXA13* gene and a prostate cancer GWAS identified a risk region form a repressive chromatin loop. When the risk locus is deleted, the loop is disrupted, resulting in *HOXA13* expression and genome-wide changes in the transcriptome, including overexpression of an oncogene (*GATA2*) previously shown to be associated with prostate cancer [154], as shown in Fig. 4B. In bladder cancer, the luminal-papillary subtype and basal/squamous subtype have special chromatin loops that can regulate key oncogenes in each subtype [155]. Research combining Hi-C and RNA-seq reveal that enhancer-promoter loops containing colorectal cancer-specific enhancers are involved in changes in 50% of TADs and interact with 152 highly expressed genes, including *ITGB4*, *RECQL4*, *MSLN*, and *GDF15*, in colorectal cancer cells compared to normal colon cells, which may play an important role in the progression of colorectal cancer [156].

Hi-C and other similar methods can also be applied to cancer to detect genomic variation. In 2017, Hi-C was used for the first time to detect chromosome rearrangements and CNV in human tumors [157]. The new algorithm developed in the following year was used to detect whole genome SV [158]. Various types of SV can be comparatively observed on the contact diagram of Hi-C [159]. A Hi-C heatmap can show the detailed changes in chromosomes by breaking and remapping, and it can even be seen that the broken ends overlap with SV [160, 161].

## 3D genome disorganization in congenital diseases

3D genome disorganization has been observed in congenital diseases. Here, we introduce these changes.

**Figure 5.**
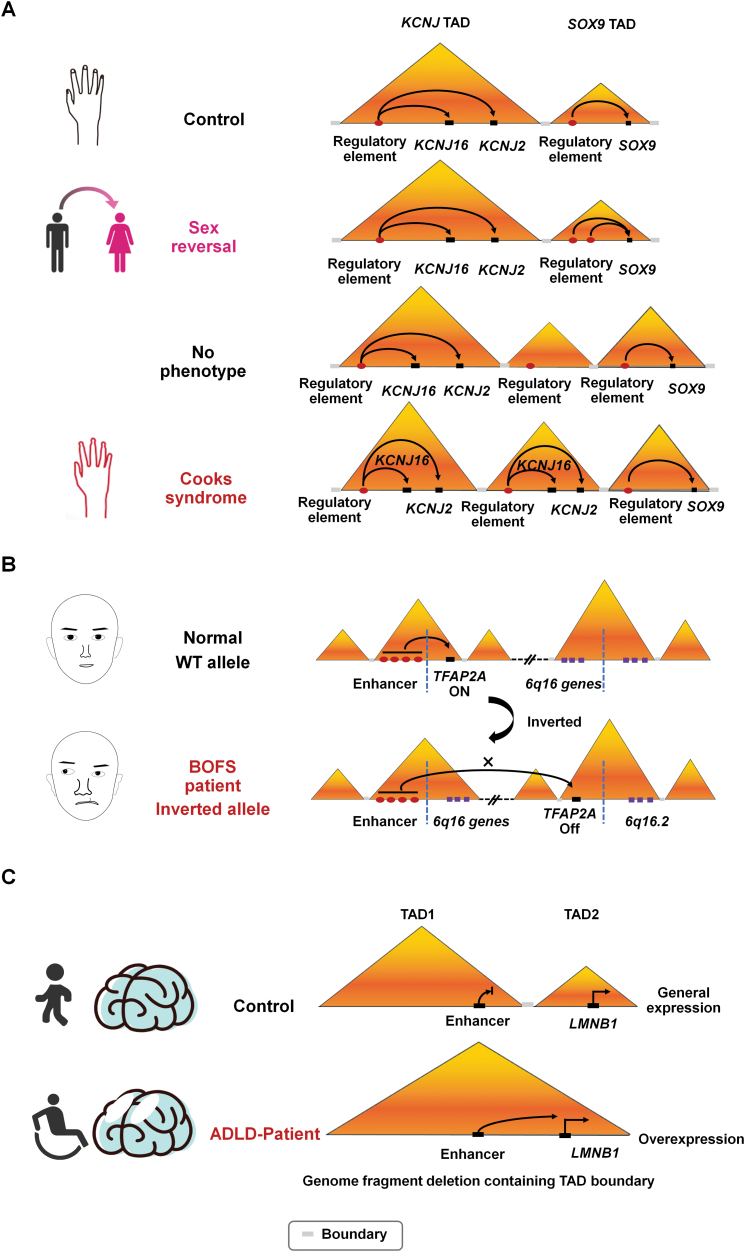
Genome disorganization in congenial diseases. **(A)** TAD disorganization in the etiology of Cooks syndrome. In cells of healthy individuals, the *SOX9* gene and its regulatory element are located in one TAD, and the other two genes, *KCNJ2* and *kCNJ16*, and their regulatory elements are located in another TAD. These two TADS are insulated from each other. Sex reversal: when the chromatin fragment of the regulatory element in the *SOX9* gene TAD is duplicated, the phenotype is reversed from female to male. No phenotype: The chromatin fragment carried duplicated *SOX9* regulatory elements, resulting in the emergence of a new TAD, and no subsequent phenotype when the duplicated regulatory elements are located in the new TAD that separats it from the *SOX9* and *KCNJ16/KCNJ2* genes. Cooks syndrome: *KCNJ* TAD duplication with genes and regulatory elements leads to Cooks syndrome. (**B)** In a patient with BOFS, the fragment containing the *TFAP2A* locus is translocated from inside a TAD to a new location, and the original enhancer cannot interact with *TFAP2A*, preventing normal expression of *TFAP2A*. (**C)** In ADLD disease, the disappearance of the boundary between two TADs allows enhancers to act on the *LMNB1* promoter, resulting in its overexpression.

As one of the first examples showing the causal relationship between the 3D genome and congenital disease, deletions, inversions, or duplications of the TAD composing the *WNT6/IHH/EPHA4/PAX3* locus were found to lead to abnormal limb development and disease such as polydactyly in 2015 [162]. Since then, an increasing number of examples have been reported. Cooks syndrome is a malformation syndrome affecting the apical structures of digits and presenting hypo/aplasia of nails and distal phalanges. Under normal circumstances, *SOX9* and its regulatory elements are located in one TAD, and its adjacent TAD contains two genes, *KCNJ2* and *KCNJ16* (encoding two potassium channel proteins), and their regulatory elements. These genes are expressed independently under normal conditions, and the two TADs do not interfere with each other. However, a new TAD (neo-TAD) appears to contain regulatory elements of the *SOX9* gene and a copy of the *KCNJ2* gene, which results in abnormal *KCNJ2* expression and malformations associated with Cooks syndrome. In the same region, a sex reversal phenotype occurs when the fragment containing the regulatory element is duplicated within the *Sox9* TAD. In contrast, boundary element repeats forming a neo-TAD isolated from the rest of the genome show no phenotype [163] (Fig. 5A). Another example is the ectopic expression of the *sonic hedgehog* gene induced by enhancer adoption [164]. There is also a case of TAD boundary disruption in Liebenberg syndrome, a congenital autosomal dominant upper extremity malformation that results in ectopic activation of enhancers and misexpression of the *PITX1* gene [165].

Both overexpression and underdose of *FOXG1* are related to chromosomal rearrangements, leading to diseases such as Rett syndrome that seriously affect children’s psychomotor development [166]. Mutations in the neural crest regulator gene *TFAP2A* can cause a rare genetic disorder, branchiooculofacial syndrome (BOFS), whose patients have different facial and ocular appearances [167]. One BOFS patient shows chromosome inversion, where the *TFAP2A* gene is transferred to another TAD and separated from its original enhancer that is located in the same TAD as the *TFAP2A* locus in the WT allele, resulting in the underexpression of the *TFAP2A* gene [168] (Fig. 5B). More than 25 genetic diseases, such as fragile X syndrome, Huntington’s disease, and amyotrophic lateral sclerosis, have been reported to be caused by unstable expansion of short tandem repeats (STRs), these pathogenic STRs are all in the boundary of TAD or sub-TAD with high CpG island density. In healthy individuals, the *FMR1* gene is normally expressed at the TAD boundary. In fragile X syndrome, the STRs within the *FMR1* gene are unstable and expand with DNA methylation so that CTCF cannot bind, resulting in silencing of the *FMR1* gene [169]. In a rare neurological condition, autosomal dominant adult-onset demyelinating leukodystrophy (ADLD), the disappearance of the TAD boundary is thought to occur via an enhancer adoption mechanism and results in *LMNB1* gene overexpression [170], as shown in Fig. 5C. In addition, it has been shown that 7.3% of TAD boundaries are disrupted, which contain known syndromic loci, in human congenital balanced chromosomal abnormalities [171].

## 3D genome disorganization in virus infection

The outbreak of COVID-19 reminds us to pay more attention to the relationship between the virus and human health and their impact on the spatial structure of the host genome [172]. Here, we focus on several widely concerning changes in the host genome before and after viral infection.

**Figure 6.**
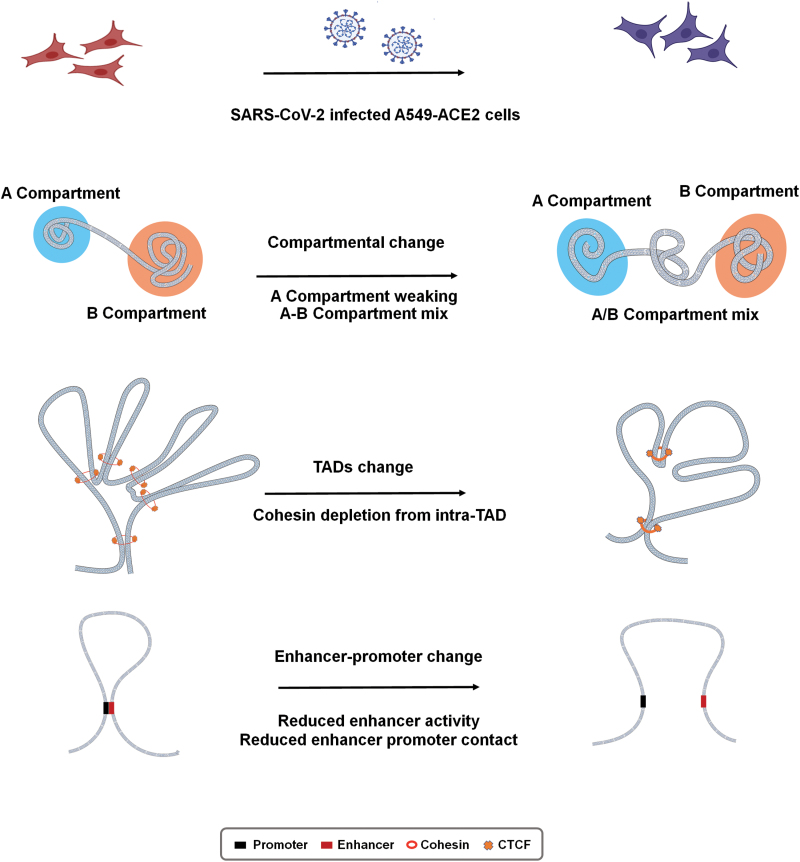
The 3D genome restructures in the host chromatin architecture after SARS-CoV-2 infection. SARS-CoV-2 infection was performed in A549 cells expressing the ACE2 receptor (A549-ACE2), and the changes in the 3D architecture of chromosomes before and after infection were analyzed using *in situ* Hi-C. First, principal component analysis shows that the chromatin contact in the A compartment was weakened and that the A–B mixed compartment phenomenon appeared. Second, the chromatin structure was examined at a fine scale, the content of cohesin in TAD was significantly reduced, and the frequency of chromatin contact in TAD decreased. Finally, enhancers and promoters have changes that are associated with the interferon response and transcriptional dysregulation of inflammatory genes.

The pathogen causing the COVID-19 outbreak is SARS-CoV-2. Hi-C analysis of uninfected and SARS-CoV-2-infected A549 cells expressing the human SARS-CoV-2 entry receptor angiotensin-I-converting enzyme 2 receptor (ACE2) showed that after 24 h of infection, the whole compartments of A549 cells redistribute, which corresponds well with the H3K27ac signal changes [173]. High-resolution Hi-C analysis also reveals reduced intra-TAD contacts, depletion of cohesin complexes in the TAD region, emergence of extremely long-distance intra- and inter-chromosomal interactions, and changes in chromatin loops, which are associated with the downregulation of the IFN gene (*DDX58*) and upregulation of the inflammatory factor (*IL6*) [174], after SARS-CoV-2 infection (Fig. 6).

Hepatitis B virus (HBV) is one of the major risk factors for hepatocellular carcinoma. HBV infects human hepatocytes to form covalently closed circular DNA molecules and integrate HBV DNA into the host genome. Combined Hi-C and viral DNA capture (CHi-C) in primary human hepatocytes infected with HBV showed that HBV preferentially contacts CpG islands, which are often associated with highly expressed and deregulated genes during HBV infection [175]. The 3C-high-throughput genome-wide translocation sequencing (3C-HTGTS) method showed that H3K4me1-rich regions modified by KMT2C/D in the host genome interact with HBV DNA [176]. Moreover, the transcriptionally inactive positions in the HBV covalently closed circular DNA molecules tend to be close to the B compartment of chromosome 19 rather than randomly distributed [177]. In addition, HBV X protein (HBx) mediates HBV covalently closed circular DNA that correlates well with actively transcribed host chromatin [178]. YY1 and HBx enhance HBV covalently closed circular DNA transcription through host enhancers [179]. Frequent chromosomal rearrangements have also been found at HBV integration sites, associating with changes in cancer driver genes located distantly [180].

Epstein‒Barr virus (EBV) was the first discovered human tumor virus. Hi-C analysis of chromatin structure of gastric cancer cells after EBV infection reveals that the host chromatin undergoes a heterochromatin to euchromatin transition. EBV reprograms the enhancers of the host H3K9me3 heterochromatin region, thereby activating the expression of nearby proto-oncogenes and promoting tumor development [181]. *In situ* Hi-C analysis in Daudi, KemIII, RaeI, Raji, and Burkitt lymphoma cells infected with EBV episomes (latent EBV) showed that how EBV episomes interact with the host genome depends on chromosome gene density [182]. The contact loci between EVB episomes and the host genome are augmented in super enhancers that are enriched for transcription cofactors or mediators that regulate B-cell growth, enhance cell proliferation, and promote viral replication [183]. Moreover, the EVB episome preferentially interacts with the repressive histone mark H3K9me3 and the cellular genomic locus of AT-rich flanking sequences, which correspond to genes with latent transcriptional silencing [184].

Cervical cancer is a very common type of cancer in women worldwide [185]. The main causative factor is human papillomavirus (HPV) infection, and one of the carcinogenic mechanisms is that HPV DNA frequently integrates into the human genome [186] and genomic structural alterations at HPV insertion sites [187]. In one of the cell models of cervical cancer, HeLa cells, a long-range chromatin interaction between the integrated HPV fragment and the *MYC* gene and the 8q24.22 region was detected by 3C [186]. HPV integration site capture technology showed that an integrated viral genome forms loops with the host genome, and local disruption drives host gene dysregulation [188]. The newly identified integration hotspot *CCDC106* gene on chromosome 19 alters the local chromosome structure of TAD, which downregulates the tumor suppressor *PEG3* [189]. Subsequent research showed that HPV integration also promotes cervical cancer by inducing SV in the human genome [190].

## Conclusions and future prospects

**Figure 7.**
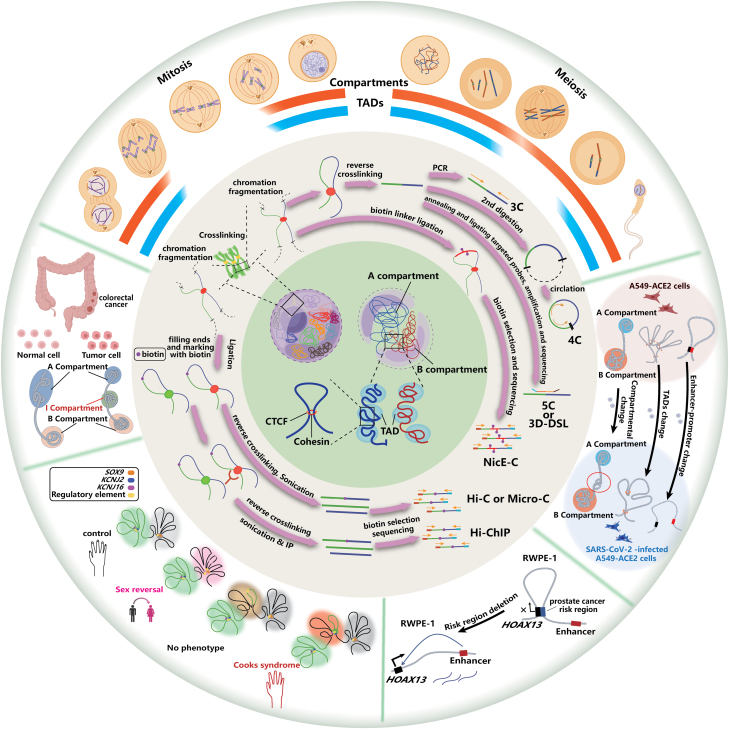
A model showing 3C-based technologies and 3D genome organization in normal physiology and disease processes.

Our understanding of the hierarchical spatial organization of genomes in cells has increased alongside the successful development and application of a suite of bioanalytical methods that provide users with access to these diverse levels, from the initial 3C one-to-one, to the subsequent 4C one-to-all, 5C- and 3D-DSL many-to-many, to general genome-wide Hi-C, to focused genome-wide Capture Hi-C, NicE-C, and HiCAR and to protein-mediated interactions ChIA-PET, PLAC-seq, and HiChIP (Fig. 7). Among different 3C-based techniques, Hi-C is the most commonly used to reveal 3D genome structure, but it lacks a higher resolution to see smaller-scale interactions, such as enhancer-promoter loops and sub-TADs. Micro-C can reach high resolution; however, it requires at least 10-fold higher sequencing depth than Hi-C; thus, the expense burden is high. Some methods have solved these problems by focusing on certain sequences of the genome, such as open chromatin region interactions. For example, NicE-C and HiCAR are especially suitable for detecting enhancer-promoter loops, with high resolution (to Micro-C level) and low sequencing depth and cost (to the normal Hi-C level), yet at the same time can detect different chromatin structures.

The disruption, reproduction, and rearrangement of compartments, TADs, and loops are closely related to specific gene expression [9, 191–193]. However, the clear relationship between 3D genome structure and transcription is not very clear. New techniques and special biological processes that are uniquely suited for studying this relationship are needed. Such a process usually involves strong 3D genome structure changes, such as mitotic metaphase and prometaphase of meiosis I, where both TADs and loops disappear but transcription status is different, and when cells go from metaphase to G1. In addition, the functions of corresponding proteins (lncRNAs) may be completely different in cell populations and individual single cells. For example, after cohesin depletion, TAD-like structures still exist at the single-cell level, while TADs almost disappear at the bulk-cell level [194].

We need to be aware that disrupting some TADs leads to dramatic expression changes in some genes; however, others show no difference. This means that there are different subtypes of TADs; some may be more structural and more stable in different conditions and processes, yet others may be more variable and involved in regulatory functions. All these results indicate the complexity of chromatin structure composition and regulatory mechanisms, and follow-up exploration still has a long way to go.

In addition to applying Hi-C-related methods to diseases, we can also analyze some existing data again to obtain new insights. For example, genome-wide association studies can determine the sequence variation in the whole human genome and screen out the single nucleotide polymorphisms (SNPs) related to diseases [195]. However, most SNPs are in the noncoding region, and most of them overlap with DNase I hypersensitivity sites. It is speculated that they might be regulatory elements [196]. Based on 3C-based technology, linking SNPs to genes of interest, we may be able to obtain a clearer picture of the GWAS results. For instance, 3C-based technologies have been used to identify chromatin loops between SNPs associated with cancer risk and their target genes found in GWAS in some malignant tumors [197, 198]. Beyond SNPs in the noncoding region, a large underdeveloped area is the lncRNA function in 3D genome structure and gene regulation (see excellent reviews on this topic for details in [35, 199]).

It is speculated that structural changes in the 3D genome will ultimately lead to differential gene expression, and 3D genome technologies can detect more upstream changes in transcriptional regulation. After understanding the structural changes within and between chromatin, we may find appropriate therapeutic targets according to these changes and develop ways to regulate the interaction of chromatin for treating diseases. However, there are still limitations in their applications, such as the limited starting materials and low resolution for specific cell types. We expect more advanced and specialized technologies to be developed, which will more widely open up the 3D genome structures field and their applications in human health and disease (Fig. 7).
